# Paediatric meningococcaemia in northwestern Ontario, Canada: a case for publicly funded meningococcal B vaccination

**DOI:** 10.1099/jmmcr.0.005017

**Published:** 2016-02-06

**Authors:** Vic Eton, Raymond S. W. Tsang, Marina Ulanova

**Affiliations:** ^1^​ Northern Ontario School of Medicine, Thunder Bay, Ontario, Canada; ^2^​ Vaccine Preventable Bacterial Diseases, National Microbiology Laboratory, Winnipeg, Manitoba, Canada; ^3^​ Lakehead University, Thunder Bay, Ontario, Canada

**Keywords:** amputation, Canada, cefotaxime, ceftriaxone, meningococcaemia, *Neisseria meningitidis*, Ontario, purpura fulminans, serogroup B, vancomycin

## Abstract

**Introduction:**

*Neisseria meningitidis* serogroup B is an important infectious agent in developed countries, including Canada. Infants are particularly susceptible to infection with serogroup B because of immature immune systems, pathogen virulence factors and changing serogroup dynamics in the post-vaccination era. Currently, the Ontario provincial government does not include serogroup B in its routine publicly funded meningococcal vaccination program.

**Case Presentation:**

A formerly well 14-month-old male presented to a tertiary hospital emergency department with fever, minor respiratory problems, diffuse purpuric rash, distended abdomen, tachycardia, and history of one episode of vomiting and melena each. Meningococcaemia was immediately suspected, and he was treated with ceftriaxone, cefotaxime and vancomycin before transfer to a different acute care facility within 12 h. *N. meningitidis* serogroup B, sensitive to ceftriaxone and penicillin, was identified in his blood. The patient developed gangrene of the lower legs and underwent bilateral below-knee amputation 8 days post-admission.

**Conclusion:**

This instance of meningococcaemia with extensive sequelae is an example of the various serious outcomes of meningococcal infection. It provides persuasive reason for routine publicly funded vaccination against *N. meningitidis* serogroup B in Ontario.

## Introduction

*Neisseria meningitidis* is a Gram-negative diplococcus and obligate human pathogen of which there are 12 serogroups, but only six (A, B, C, W-135, X and Y) produce high morbidity and mortality (Pace & Pollard, 2012; Stephens & Greenwood, 2007). Depending on factors such as age and living environment, asymptomatic nasopharyngeal *N. meningitidis* carriage rates reportedly range from 5 to 10 % in the general US adult population, but can be as high as 34 % at UK universities (MacNeil & Cohn, 2011; Neal *et al.*, 2000). *N. meningitidis* is a global causative agent of paediatric meningitis and septicaemia, and there are ∼200 cases of invasive meningococcal disease reported per year in Canada (Andrews & Pollard, 2014; Public Health Agency of Canada, 2014). Meningococcal infection is notoriously difficult to recognize early, and is associated with rapid escalation and severe sequelae; a British study reported resultant major disability rates of ∼10 % (Viner *et al.*, 2012).

As noted by Dang *et al.* (2012), serogroup B currently causes most cases of invasive meningococcal disease in Ontario, Canada, and infants are at highest risk. Since beginning the current routine publicly funded infant vaccination program against *N. meningitidis* serogroup C in 2004, the incidence of meningococcal serogroup C infection in Ontario has decreased substantially without evidence of serogroup replacement (Bettinger *et al.*, 2009; Le Saux *et al.*, 2009). Nonetheless, since the overall number of cases of invasive meningococcal disease has decreased with the widespread administering of serogroup A, C, Y and W-135 vaccines as of 2009, the proportion attributable to serogroup B has increased in Canada to ∼70 % in children younger than 5 years old (Salvadori & Bortolussi, 2011). This is part of a global trend currently observed in developed nations with publicly funded immunization programs (Gounder *et al.*, 2015; Khatami & Pollard, 2010).

The multicomponent meningococcal B (4CMenB; Bexsero) vaccine includes three recombinant proteins as well as outer membrane vesicles developed from meningococcal NZ98/254 strain (Carter, 2013). In phase IIb or III trials, a majority of infants developed protective serum bactericidal antibody against *N. meningitidis* serogroup B strains after three doses of 4CMenB administered when they were less than 6 months of age (Carter, 2013). It is hitherto unknown how 4CMenB performs at a population level (National Advisory Committee on Immunization, 2014).

As of 2015, the Ontario provincial publicly funded immunization schedule lists 12 months as the age for routine meningococcal C conjugate vaccination, with meningococcal conjugate ACYW-135 advised for administration in grade 7 (typically age 12–13 years). Licensed in Canada since late 2013, 4CMenB vaccine is presently publicly funded only for those aged 2 months to 17 years who meet high-risk criteria. This includes individuals with complement, properdin, factor D or primary antibody deficiencies; human immunodeficiency virus infection; acquired complement deficiencies; functional or anatomical asplenia; or cochlear implants (Crowcroft *et al.*, 2015; Ontario Ministry of Health and Long-Term Care, 2015). As happened in the past with other now-common and successful vaccines, the cost-effectiveness of routine 4CMenB vaccination in Ontario has been challenged (Tu *et al.*, 2014). However, there is significant support for its incorporation into national childhood immunization programs in other countries (Joint Committee on Vaccination and Immunisation, 2014; The Lancet Infectious Diseases, 2014).

## Case report

A previously well 14-month-old Aboriginal (Métis) male presented at a regional tertiary hospital emergency room with history of 3 days of fever (38–39 °C) and minor respiratory tract problems. He experienced one vomiting and one melena episode; a diffuse purpuric rash appeared on the third day of fever. He was lethargic with diminished appetite, decreased urine output and a distended abdomen.

The patient was immediately admitted to hospital. He was administered bolus fluid at 20 ml kg^− 1^ over 15 min, ceftriaxone, vancomycin and dexamethasone. Upon physical examination, he was found to have a heart rate of 205–210 beats min^− 1^ and a respiration rate of 38 breaths min^− 1^; his arterial O_2_ saturation was 98 % on room air. Arterial blood gases were low, i.e. pH 7.31, pCO_2_ 29 mmHg, pO_2_ 31 mmHg, HCO_3_ 15 mmol l^− 1^, total CO_2_ 16 mmol l^− 1^, venous O_2_ saturation 52 % on room air and base excess − 10.3 mmol l^− 1^. Sodium (133 mmol l^− 1^), potassium (3.2 mmol l^− 1^) and bicarbonate (15 mmol l^− 1^) were also low, whilst lactic acid was high (5.6 mmol l^− 1^). Partial thromboplastin time and international normalized ratio were high (64 seconds and 2.3, respectively), as were white blood cell (15.2 × 10^9^ l^− 1^), monocyte (1.3 × 10^9^ l^− 1^), band neutrophil (2.3 × 10^9^ l^− 1^), metamyelocyte (0.8 × 10^9^ l^− 1^) and myelocyte (0.6 × 10^9^ l^− 1^) counts. He had low haemoglobin (104 g l^− 1^), haematocrit (31 %), platelet (96 × 10^9^ l^− 1^) and lymphocyte (1.1 × 10^9^ l^− 1^) counts. A nasogastric tube was placed *in situ* and he underwent tracheal intubation. He was admitted to the intensive care unit with a presumptive diagnosis of meningococcaemia and likely sepsis.

His perinatal history was unremarkable. He was born via spontaneous vaginal delivery at 37 weeks 3 days gestation; Apgar score of 9 at 1 and 5 min; had a birth weight of 2999 g; and tested negative for sickle-cell disease, congenital adrenal hyperplasia, severe combined immunodeficiency, cystic fibrosis and haemolytic anaemia. He was discharged from hospital 2 days after birth. Prior to this incident, he had no significant hospitalizations on record, although he had received same-day emergency attention for a respiratory viral infection 2 months earlier.

An electrocardiogram revealed sinus tachycardia with short PR interval, non-specific intra-ventricular conduction block and T-wave inversion in inferior leads. Chest X-rays revealed mild bilateral perihilar haziness with parabronchial thickening, whilst abdominal X-rays and ultrasound found nothing significant.

### Diagnosis

*N. meningitidis*, sensitive to ceftriaxone and penicillin, was cultured from the patient's blood and identified as serogroup B using standard methodology. Identification of serotype and serosubtype, clonal analysis, and determination of PorA genotype as well as of the *fHbp*, *nhba* and *nadA* gene sequences were done as described previously (Law *et al.*, 2015). Ten hours after admission to the intensive care unit, the patient was transferred from the initial receiving hospital for care at another acute facility with a diagnosis of meningococcaemia.

### Treatment

Prior to transfer, the infection was treated with ceftriaxone sodium (two discontinuous intravenous doses of 1 g within 10 min), cefotaxime sodium (one discontinuous intravenous dose of 1 g and an intravenous dose of 0.9 g every 6 h over 9 h) and vancomycin-HCl (one intravenous dose of 180 mg every 6 h over 11 h and two discontinuous intravenous doses of 500 mg 6 h apart).

### Outcome and follow-up

Extensive purpura fulminans and consequent tissue necrosis affected the patient's lower legs and right arm. One week after his meningococcaemia diagnosis, he underwent bilateral below-knee amputations to manage gangrene. He was deemed to be healing and coping well with no apparent further sequelae 7 months after his amputations.

The *N. meningitidis* isolate was characterized as follows: serotype 4; serosubtype P1; – (non-serosubtypable); PorA genotype P1.21, 16-36, 37-1; multilocus sequence type ST-3327, which belongs to the ST-885 clonal complex. The 4CMenB vaccine antigen gene characteristics of the isolate were as follows: factor H binding protein (*fHbp*) predicted to produce peptide 108 (gene allele 108) belongs to the vaccine variant 1; *Neisseria* heparin binding antigen (NHBA) predicted to produce peptide 24 (*nhba* gene allele 15); NadA was absent.

Following laboratory investigation for possible underlying immune deficiency, the patient was revealed to be IgA-deficient, but otherwise immunocompetent.

### Ethical statement

The Thunder Bay Regional Health Sciences Centre and Lakehead University research ethics boards in Ontario, Canada, approved this study.

## Discussion

Meningococcaemia is the distribution of *N. meningitidis* in the bloodstream, which begins following the adhesion of meningococci to nasopharyngeal epithelial cells. The bacterium's manufacture of IgA protease, production of factors hindering ciliary activity and polysaccharide capsule that inhibits opsonophagocytosis all contribute to its virulence (Pathan *et al.*, 2003). The capsular polysaccharide of *N. meningitidis* serogroup B is antigenically similar to the human neural cell adhesion molecule and does not consequently induce adaptive immune response (Pathan *et al.*, 2003). Meningococcaemia is associated with very high quantities of endotoxin in the host's bloodstream (Andersen, 1989). The circulating endotoxin's interaction with the innate immune system triggers severe inflammation (Pathan *et al.*, 2003). Purpura fulminans is an extremely serious consequence of meningococcaemia caused by microvascular injury accompanied by extensive coagulation in small blood vessels within the skin and extreme thrombocytopenia; it can result in thrombosis and haemorrhagic necrosis, and gangrene, disseminated intravascular coagulation and multi-organ failure may follow (Jacobsen & Crawford, 1984; Levi *et al.*, 1999; Pathan *et al.*, 2003).

In children less than 2 years old, immature immune responses render them especially unequipped to contend with encapsulated bacteria, such as *N. meningitidis* (Riordan, 2014; Stephens *et al.*, 2007). Passively transferred maternal IgG confers protection during the first months of age, but the levels drop off whilst production of bactericidal antibodies slowly increases with age.

For those cases of invasive meningococcal disease occurring in the first year of life, ∼70 % occur in infants younger than 6 months of age (Dang *et al.*, 2012; Bettinger *et al.*, 2013). Meningococcaemia is a critical condition, and can result in substantial morbidity and mortality if not promptly treated (Dashefsky *et al.*, 1983). Robinson (2014) identified amputation, scarring, renal dysfunction, deafness and neurological sequelae (including seizures and paralysis) as possible outcomes for infection with meningococcal serogroup B. Incidence of dramatic sequelae was found to be greatest (26–27 %) in children less than 4 years of age (Robinson, 2014).

Multiple risk factors may have contributed to this patient's susceptibility to the condition. Second-hand smoke exposure reportedly more than doubles the risk of invasive meningococcal disease, perhaps related to the immunosuppressive effects of nicotine (Lee *et al.*, 2010; Sopori, 2002). Whilst the patient's mother did not smoke throughout her pregnancy, nothing is known about her habits following delivery or the habits of the two other adults in the household. As the patient was first admitted for infection in late December, season (and the behaviours and exposures associated therewith) may have also influenced his susceptibility to infection. Kinlin *et al.* (2009) report that season, particularly late winter and early spring, was correlated with higher rates of invasive meningococcal disease in a North American city. Infection with influenza A and respiratory syncytial viruses, independent of season, may also increase the risk of invasive meningococcal disease (Tuite *et al.*, 2010). Notably, the patient was treated for respiratory viral infection 2 months prior to developing meningococcaemia and experienced minor respiratory tract problems at the time of diagnosis. Myriad genetic determinants, particularly complement deficiencies and defects in innate immune receptor signalling or opsonophagocytosis, may underlie host susceptibility to meningococcal infection (Emonts *et al.*, 2003). Although the patient was IgA-deficient, such immunodeficiency is not reportedly associated with an increased susceptibility to invasive bacterial infection; most individuals with selective IgA deficiency are asymptomatic (Aghamohammadi *et al.*, 2009; Ammann & Hong, 1971; Ozkan *et al.*, 2005). However, as IgA deficiency is linked to vulnerability to recurrent respiratory infections, which are in turn associated with invasive meningococcal disease, the significance of his immunodeficiency cannot be determined unequivocally (Ozkan *et al.*, 2005; Tuite *et al.*, 2010). Lastly, the patient was Aboriginal, an ethnic group which experiences health disparities associated with social, economic, cultural and political inequities in Canada (Adelson, 2005).

Whilst the performance of the 4CMenB vaccine has not been scrutinized at the population level, a New Zealand study found that a strain-specific outer membrane vesicle vaccine against group B meningococcal disease, administered in a broad population campaign, effectively reduced the incidence of meningococcal disease due to the epidemic strain in pre-school-aged children (Galloway *et al.*, 2009). The 4CMenB vaccine can be anticipated to be similarly successful, as it contains the same antigen used in the New Zealand study (along with three other recombinant proteins) and has been demonstrated to reduce meningococcal carriage rates in clinical trials (Read *et al.*, 2014; Snape & Pollard, 2013). The UK's Joint Committee on Vaccination and Immunisation (2014) recently advised routine infant vaccination with the 4CMenB vaccine, so further data on the epidemiological efficacy of the vaccine will likely be available in the future.

Based on known characteristics of the 4CMenB vaccine, it could be protective against the infection caused by *N. meningitidis* strain in this case. In particular, an fHbp peptide 108 of the isolate belongs to the vaccine fHbp subfamily B or variant 1. As proteins within the fHbp subfamily show a good degree of similarity, cross-protection amongst these peptides is likely (Masignani *et al.*, 2003). Although NHBA peptide 24 does not directly correspond to peptide 2, which is a vaccine component, variants of NHBA may offer cross-protection that was found in animal studies (Giuliani *et al.*, 2006).

The severe outcome of this particular case parallels one described by Federico Martinón-Torres referenced in a recent Editorial in *The Lancet Infectious Diseases* and constitutes one of the severest outcomes of fulminant meningococcaemia (Canavese *et al.*, 2010; Pace & Pollard, 2012; The Lancet Infectious Diseases, 2014). It provides a compelling argument other than cost-effectiveness for the merit of routine publicly funded vaccination against *N. meningitidis* serogroup B in Ontario.
